# The Effects of Smoking on IgE, Oxidative Stress and Haemoglobin Concentration

**DOI:** 10.31557/APJCP.2020.21.4.1069

**Published:** 2020-04

**Authors:** Najat J Ahmed, Adnan Z Husen, Najmaddin Khoshnaw, Hisham A Getta, Zahir S Hussein, Ahmed K Yassin, Sana D Jalal, Rebar N Mohammed, Alaa F Alwan

**Affiliations:** 1 *Department of Medical Laboratory Technology, Health-Technical College, Erbil Polytechnic University, *; 3 *Department of Clinical Hematology, Kurdistan Board for Medical Specialties, Ministry of Higher Education and Scientific Research, *; 5 *Department of Internal Medicine, College of Medicine, Hawler Medical University, Erbil, *; 5 *2Department of Hematology, Hiwa Cancer Hospital, Sulaymaniyah, *; 4 *Department of Hematopathology, College of Medicine, *; 6 *Department of Microbiology, College of Veterinary Medicine, University of Sulaimani, Kurdistan Region, *; 7 *7Department of Clinical Hematology, National Center of Hematology, Mustansiriyah University, Baghdad, Baghdad, Iraq. *

**Keywords:** Smoking, Malondialdehyde, IgE

## Abstract

**Background:**

Smoking is a well-known related factor for many health problems in a human being through different ways of exposure.

**Objectives:**

Thie aim of the study was to examine the effects of different types of cigarette smoking on hemoglobin level, high sensitive C-Reactive Protein (hsCRP), Malondialdehyde (MDA), and IgE levels in healthy adult subjects.

**Methods:**

One hundred seventy-one healthy adult females and males were included in this study. They divided into four groups: cigarette, shisha, passive smokers, and non-smokers groups. Serum samples from all groups analyzed for hemoglobin, hsCRP, IgE, and malondialdehyde level.

**Results:**

The mean MDA, IgE, and hemoglobin levels significantly increased in both smokers (cigarette and Shisha groups) and passive smokers than in non-smokers group (p<0.05). The hsCRP levels were significantly increased (p<0.05) in cigarette and Shisha smokers compared to non-smokers. At the same time, there was a non-significant relationship between passive smoker in comparison to non-smokers (p>0.05).

**Conclusion:**

This study concluded that smoking, including cigarette and shisha, even passive smoking harmed health through increasing Malondialdehyde, serum IgE and hs-CRP levels in the body.

## Introduction

Smoking was regarded as the cause of one/third of all cancer deaths worldwide, and for nearly 80% of COPD related deaths (Kibria et al., 2017). It has been known that cigarette smoking carries around 7,357 chemical compounds, including toxic metals, poisonous gasses, and free radicals (Rogman and Perfetti, 2013). A statistically significant association between shisha smoking and the development of lung cancer by four-fold compared to none-smokers has been reported (Jaggi and Yadav, 2015; Kadhum et al., 2015). 

Smoking has been proved to be linked to many cancers, including lung and gastrointestinal and bladder cancers (Gandini et al., 2008). Smokers exhibit increased numbers of lung macrophages, and these cells are likely recruited to the lung to clear the particulate material associated with tobacco smoke. These macrophage dysfunctions likely contribute to the immunosuppression that is associated with tobacco and smoking. The data reported to date indicate that cigarette smoking is related to the suppression of B-cell function and immunoglobulin (Ig) production (Barbour et al., 1997). However, more recent reports suggest enhanced NK cell activity associated with active smoking in patients with chronic obstructive pulmonary disease. A remarkable association was found between tobacco smoke, as shown by serum nicotine levels and IgE sensitization with potential dose-dependent relationships (Yao et al., 2016). However, other studies reported an increased number of white blood cells (WBC) and leukocytes in response to smoking (Mirakhori, 2014). It was estimated that each puff of cigarette smoke contains about ten free radicals (Armstrong, 2012). The oxidative stress and inflammatory potential were tested in different cigarette products concerning their tar, nicotine, H_2_O_2_, and nitric oxide content. These harmful products can ultimately impair the blood-brain barrier function and increase the risk for the pathogenesis of several CNS disorders (Naik et al., 2014). High sensitive C-reactive Protein (hs-CRP) is a marker of systemic vascular inflammation that was increased in smokers has been demonstrated to have a role in predicting primary and secondary coronary events (O’Loughlin et al., 2008). Malondialdehyde (MDA) is one of the markers that is used to evaluate oxidative stress, was increased in smokers (Safyudin and Subandrate, 2016). The Aim behind our study was to examine the effects of different types of cigarette smoking on hemoglobin level, high sensitive C-Reactive Protein (hsCRP), malondialdehyde and IgE levels in healthy adult subjects.

## Materials and Methods

Our study was a case-control study in which blood samples were randomly collected from cigarette smokers, shisha smokers, those living with the smokers, and controlled group (never smoker and no passive exposure from Erbil City/KRG/Iraq. Ethical approval was obtained from Erbil Technical college ethics and scientific committee prior to commencement of the study, and participants’ consent was obtained. All groups were subjected according to the interview questionnaire to determine the demographic data and to smoke history (type, duration, and amount). Informed took from each participant. The study population was divided into four groups; Cigarette smokers, shisha smokers, passive smokers, and control groups (non-smokers. Blood samples were collected from all participants in the study. The number of each group was as follows: Group 1: fifty Cigarette smokers; group 2: 50 Shisha smokers; group 3:50 passive smokers and group 4: 21 Control (non-smokers) (Table 1). 

Blood samples were taken through venepuncture from all study participants who were fasting for the last 12hrs. Then, samples were divided into 2ml for the EDTA tube and 5ml for the white test tube, and then samples centrifuged to separate the serum. The serum of all tested subjects was divided into two test tubes. One of them was Eppendorf tube (Germany), tested directly, and the second one was frozen at -20^o^C for Total IgE (KUL/L), hsCRP mg/L, MDA (nmol/ml), and hemoglobin concentration (g/dl). The diluent solution, lysis reagent, and cleaning Orphee solution (Germany) were used for the CBC analysis. We used Alcohol 70%, Distilled water, Trichloroacetic acid powder, Thiobarbituric acid powder, BioMerieux total IgE kit (France), and Spinreact for hsCRP (Spain).

## Results

**Figure 1:**
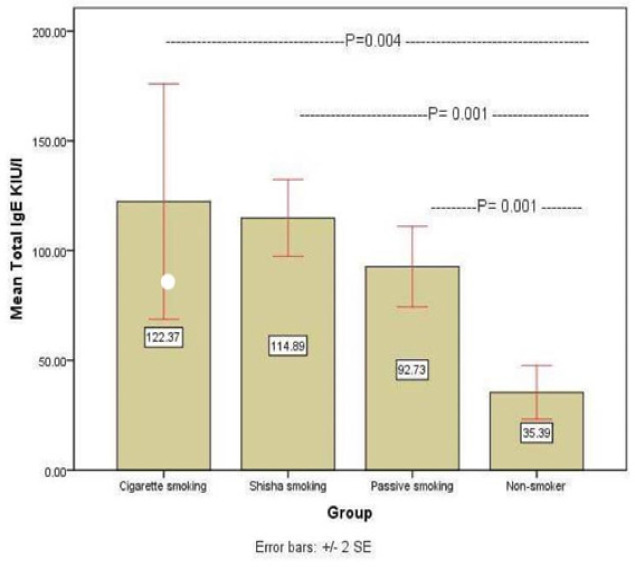
Mean Concentration of Total IgE KIU/L in sera of Different Smokers and Control Group. It shows the differences of IgE level in sera of participators. It shows that there is significant difference in the mean ± SE among tested groups (2.31 ± 0.93; p-value = 0.01, 1.57 ± 0.98).

**Figure 2:**
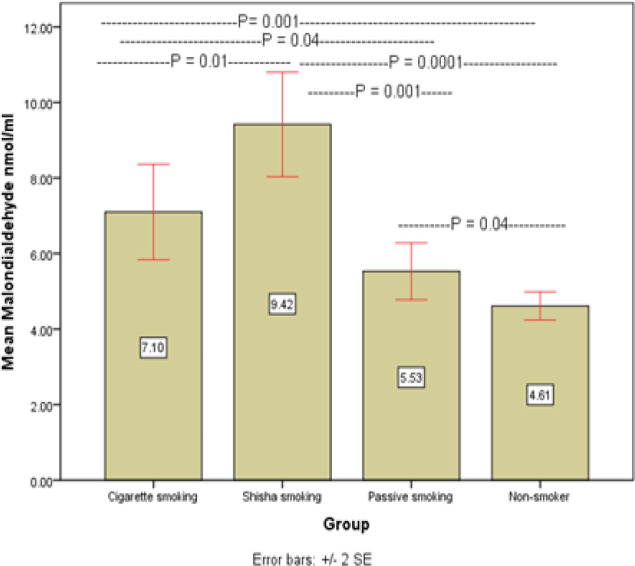
Mean Concentration of Malondialdehyde (nmol/ml) in Sera of Different Smokers and Control Group

**Figure 3:**
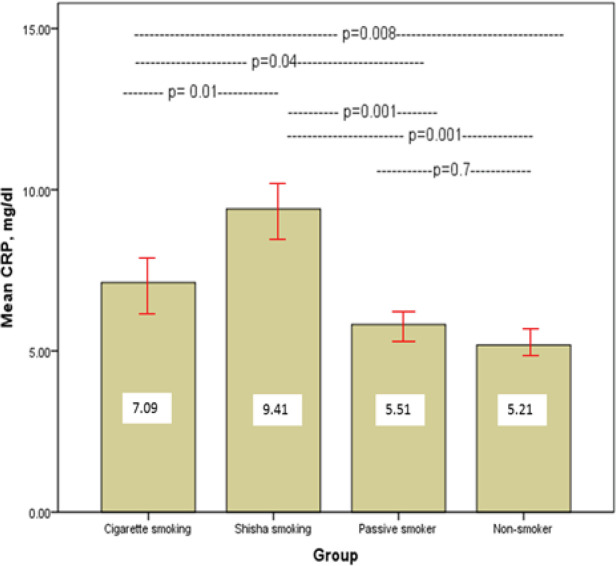
Mean Concentration hsCRP (mg/l) in Sera of Different Smokers and Control Group

We evaluated the blood of 171 normal healthy subjects, 93 ( 54.4% ) men and 78 (43.6%) women, age ranging from 20-50 years). We found an increased IgE level in smokers (Cigarette and Shesha smokers, passive) in comparison to non-smoker group, as following 122.37, 114,88, 92.73, and 35.39 kul/l respectively, (p<0.05, Table 1 and Figure 1). The mean Malondialdehyde (MDA) is significantly elevated in Shesh smokers, followed by Cigarette smokers in comparison to passive smoker non-smoker and non-smokers group (9.41, 7, 5.5, 5.2 nmol/ml) (Figure 2). The hsCRP was prominently elevated in Shesha than Cigarette smokers while no difference between the passive and non-smoker groups (9.4, 7, 5.5, 5.2 mg/l) (Figure 3) respectively.

In the other part of our study, we detected higher mean levels of the lipid profiles (total serum cholesterol, triglyceride, lower-density lipoprotein (LDL) were more in smokers (Cigarette, SHesha and passive smokers in compared to non-smokers. In contrast to that, the excellent cholesterol high-density lipoprotein (HDL) was less in the smoker group than non-smokers (Table 1).

**Figure 4:**
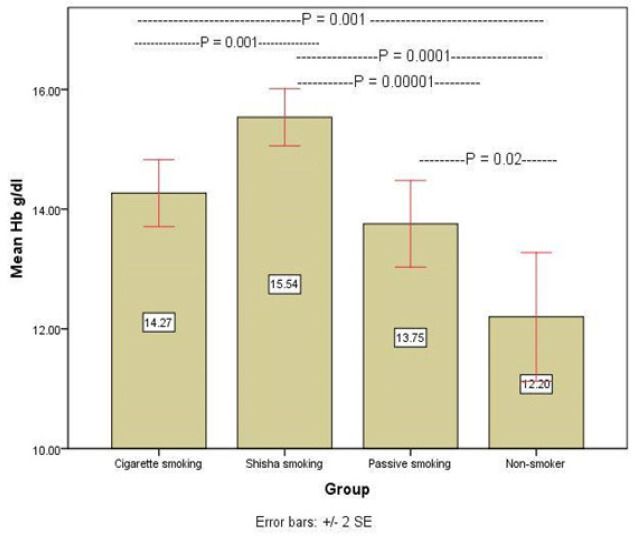
Mean Concentration Haemoglobin (g/dl) in Sera of Different Smokers and Control Group

The mean Hb concentration was more among Shesha (15.5 gm/dl) than Cigarette (14.7 gm/dl), and lowest mean Hb level found among non-smokers groups in both sexes (12.2 gm/dl) (Figure 4).

**Table 1:** Shows the Number of Participants, Gender and the Statistical Analysis for (mean ± SE), Total IgE (KUL/L), hsCRP mg/L, Malondialdehyde (nmol/ml), Total Serum Cholesterol (mg/dl), Serum Triglyceride (mg/dl), Serum LDL Cholesterol (mg/dl), Serum HDL Cholesterol (mg/dl) and Haemoglobin Concentration (g/dl).

	Gender /Number of subjects	Male: 93		
Parameters		Female: 78		
	Age / year	20 – 55		
	Groups (mean ±SE)			
	G1	G2	G3	G4
Number of cases	50	50	50	21
Total IgE kul/l	122.37 ± 26.82	114.88± 8.72	92.73 ± 9.17	35.39± 6.12
hsCRP (mg/l)	7.09± 0.63	9.41± 0.69	5.52± 0.37	5.21± 0.17
Malondialdehyde nmol/ml	7.10± 0.63	9.42± 0.69	5.53± 0.37	4.61± 0.18
Total serum cholesterol (mg/dl)	171.36± 7.84	164.76± 7.35	158.18± 11.18	141.20± 4.54
Serum triglyceride (mg/dl)	216.56± 16.30	182.68± 10.79	177.09± 12.72	157.10± 12.02
Serum LDL cholesterol (mg/dl)	96.02± 7.88	88.02± 8.92	81.25± 9.81	63.49± 3.06
Serum HDL cholesterol (mg/dl)	35.00± 1.03	35.40± 2.11	39.69± 2.12	42.29± 2.46
Hb concentration (g/dl)	14.27± 0.28	15.54± 0.23	13.75± 0.36	12.20± 0.53

G1, Cigarette smoker; G2, Shisha smoker; G3, Passive smoker; G4, Non-smoke

## Discussion

Smoking induces oxidative stress and can be measured by increased plasma malondialdehyde (Safyudin and Subandrate. 2016). Thus, the study aimed to evaluate and compare the status of oxidative stress by estimation of Malondialdehyde levels in different types of smokers and compare the risk of smoking between cigarette and shisha smokers. Smoking may increase the risk of developing allergic responses and atopic diseases and, in particular, among shisha smokers (Abdulhamid et al., 2015). The current study showed that there is no significant difference between the level of total serum IgE between cigarette and shisha smokers but had a considerable difference with non-smokers. This means that smoking followed by an increased level of IgE as an immunoglobulin stimulating allergy in smokers. This supports the existence of a link between allergic stimuli and disturbance in the defense system in smokers, as reported by Ahmedi et al., (2014) and Abdulhamid et al., (2015).

Smoking induces oxidative stress and can be measured by increased plasma Malondialdehyde. Moreover, elevated plasma levels of Malondialdehyde indicate an increase in the level of production of oxygen free radicals (Abdulhamid et al., 2015; Kalaiselvi, 2016). Thus, one of an outstanding result is the elevation of concentration of serum malondialdehyde (Lykkesfeldt et al., 2004; Rodgman and Perfetti, 2013; Kalaiselvi, 2016), but the exciting result in the current study shows that there are differences in the effect of smoke between shisha and cigarette smoker’s bodies and shows that the concentration of Malondialdehyde in the sera of shisha smokers higher than cigarette smokers. Moreover, the shisha smoke is riskier than cigarette smoke on passive smokers to produce more amount of Malondialdehyde in the body. According to the result of this study, the effect of smoke among passive smokers to elevate Malondialdehyde is clearly observed because of the concentration of Malondialdehyde in the sera of passive smokers higher than non-smokers. Our results were similar to that of Megson et al., (2013); Rodgman and Perfetti, (2013); Kalaiselvi, (2016).

On the other hand, the concentration of hemoglobin among smokers includes; passive smokers, and non-smokers show that shisha smoke is more affected than cigarettes. This finding can be seen clearly through the highly significant differences in the concentration of hemoglobin among all types of smokers and non-smokers. The former means that the smoke of shisha is more dangerous than cigarettes, even on passive smokers. This result entirely agrees with a study published by Abdulhamid et al., (2015).

Although related to inflammatory markers in adults, little is known about the association between cigarette smoking and hsCRP in smokers. The results of the current study showed a relationship between tobacco and hsCRP. Our results confirm that the tissue damage related to cigarette smoking and may begin soon after tobacco use. It was mentioned that no level or type of tobacco is safe on the body’s health (O’Loughlin et al., 2008; Desai, 2016), while Aldaham et al., (2015) stated that hsCRP is not related to smoking.

Another risk for smokers is getting infections through using of another risk for smokers is getting infections through using of mouthpieces, various commensal and pathogenic organisms might be transmitted between the smokers through saliva. Therefore, increased risk of hepatitis, herpes, and tuberculosis infection by sharing mouthpieces have been recorded (Kadhum et al., 2015). In our study, we observed that the use of a disposable mouth-piece for shisha per each individual would remarkably decrease the incidence of infection. Many researchers have noticed that shisha cafes provide a plastic disposable mouthpiece to every customer, which aims to limit the spread of infectious diseases. The current study concluded that smoking considers as a factor that leads to an immunoglobulin stimulating allergy, and shisha smoke is risky more than cigarette smoke that triggers free radicals even for passive smokers. We think our study is important for our region as cancer became a major problem in Kurdistan region, especillay tobacco related cancer as publishesd by Khoshnaw et al., (2016). 

In conclusion, this study concluded that smoking, including cigarette and shisha, even passive smoking hurt health through increasing Malondialdehyde, serum IgE and hs-CRP levels in the body.

## References

[B1] Abdulhamid RE, Sharief AH, Omer SA (2015). Association of smoking and IgE levels among smoker women in Khartoum. Am J Res Com.

[B2] Ahmadi M, Shadmehri S, Naji M (2014). Antioxidative and Allergic Profile in Adult Men with Cigarette Smoking. Biol Forum.

[B3] Aldaham S, Foote JA, Chow HH (2015). Smoking status effect on inflammatory markers in a randomized trial of current and former heavy smokers. Int J Inflam.

[B5] Barbour SE, Nakashima K, Zhang J (1997). Tobacco and smoking: environmental factors that modify the host response (immune system) and have an impact on periodontal health. Crit Rev Oral Biot Med.

[B6] Desai SV (2016). Tobacco chewing and smoking -risk for renal diseases. Biomed Res.

[B7] Gandini S, Botteri E, Iodice S (2008). Tobacco smoking and cancer: A meta-analysis. Int J Cancer.

[B8] Jaggi Sh, Yadav AS (2015). Increased serum malondialdehyde levels among cigarette smokers. Pharm Innov J.

[B9] Kadhum M, Sweidan A, Jaffery AE, Al-Saadi A, Madden B (2015). A review of the health effects of smoking shisha. Clin Med.

[B10] Kalaiselvi K (2016). Study of plasma levels of Malondialdehyde as an oxidative stress marker,” so it comes as a marker in smokers & non-smokers. PARIPEX-Ind J Res.

[B11] Kibria MG, Islam AM, Rahman MM (2017). Prevalence and determinants of smoking among young age male patients coming at OPD in selected Hospital. Med Today.

[B12] Khoshnaw N, Mohammed HA, Abdullah DA (2015). Patterns of cancer in Kurdistan - results of eight years cancer registration in Sulaymaniyah Province-Kurdistan-Iraq. Asian Pac J Cancer Prev.

[B13] Lykkesfeldt J, Viscovich M, Poulsen HE (2004). Plasma Malondialdehyde is induced by smoking: a study with balanced antioxidant profiles. Br J Nutr.

[B14] Megson IL, Haw SJ, Newby DE (2013). Association between exposure to environmental tobacco smoke and biomarkers of oxidative stress among patients hospitalized with acute myocardial infarction. PLoS One.

[B15] Mirakhori FM (2014). Lipid profile and chronic exercise training in Cigarette smoke. Biol Forum.

[B16] Nagaraj SKD, Paunipagar PV (2014). Study of serum malondialdehyde and vitamin c in smokers. J Sci In Res.

[B17] Naik P, Fofaria N, Prasad S (2014). Oxidative and pro-inflammatory impact of regular and de-nicotinized cigarettes on the blood-brain barrier and endothelial cells: is smoking reduced or nicotine-free products safe?. BMC Neurosci.

[B18] O’Loughlin J, Lambert M, Karp I (2008). Association between cigarette smoking and C-reactive Protein in a representative, population-based sample of adolescents. Nicotine Tob Res.

[B20] Safyudin S, Subandrate S (2016). Smoking tends to decrease glutathione and increase malondialdehyde levels in medical students. Universa Med.

[B21] Yao TC, Chang SW, Hua MC, et.al (2016). Tobacco smoke exposure and multiplexed immunoglobulin E sensitization in children: a population-based study. Allergy.

